# Macrocyclic Compounds: Metal Oxide Particles Nanocomposite Thin Films Deposited by MAPLE

**DOI:** 10.3390/ma16062480

**Published:** 2023-03-21

**Authors:** Marcela Socol, Nicoleta Preda, Carmen Breazu, Andreea Costas, Oana Rasoga, Gabriela Petre, Gianina Popescu-Pelin, Sorina Iftimie, Andrei Stochioiu, Gabriel Socol, Anca Stanculescu

**Affiliations:** 1 National Institute of Materials Physics, 405A Atomistilor Street, 077125 Magurele, Romania; 2 Faculty of Physics, University of Bucharest, 405 Atomistilor Street, 077125 Magurele, Romania; 3 National Institute for Lasers, Plasma and Radiation Physics, 409 Atomistilor Street, 077125 Magurele, Romania

**Keywords:** MAPLE, nanocomposite films, metal oxide nanoparticles, BHJ, optoelectronic applications

## Abstract

Nanocomposite films based on macrocyclic compounds (zinc phthalocyanine (ZnPc) and 5,10,15,20-tetra(4-pyridyl) 21H,23H-porphyrin (TPyP)) and metal oxide nanoparticles (ZnO or CuO) were deposited by matrix-assisted pulsed laser evaporation (MAPLE). 1,4-dioxane was used as a solvent in the preparation of MAPLE targets that favor the deposition of films with a low roughness, which is a key feature for their integration in structures for optoelectronic applications. The influence of the addition of ZnO nanoparticles (~20 nm in size) or CuO nanoparticles (~5 nm in size) in the ZnPc:TPyP mixture and the impact of the added metal oxide amount on the properties of the obtained composite films were evaluated in comparison to a reference layer based only on an organic blend. Thus, in the case of nanocomposite films, the vibrational fingerprints of both organic compounds were identified in the infrared spectra, their specific strong absorption bands were observed in the UV–Vis spectra, and a quenching of the TPyP emission band was visible in the photoluminescence spectra. The morphological analysis evidenced agglomerated particles on the composite film surface, but their presence has no significant impact on the roughness of the MAPLE deposited layers. The current density–voltage (J-V) characteristics of the structures based on the nanocomposite films deposited by MAPLE revealed the critical role played by the layer composition and component ratio, an improvement in the electrical parameters values being achieved only for the films with a certain type and optimum amount of metal oxide nanoparticles.

## 1. Introduction

The research interest on nanocomposites materials has increased dramatically over the years, taking into account the fact that they can be used as functional nanomaterials that are easily integrated into different areas, such as optoelectronics, environmental applications, medicine, etc. [1,2,3,4]. Organic–inorganic composites have attracted special attention due to the possibility of fabricating nanomaterials with enhanced properties by combining the features of constituent components [2,5,6].

Lately, a hot research topic has consisted of designing and developing organic–inorganic nanocomposites as thin films with tailored properties, owing to their wide range of applications in fields such as optoelectronics, sensing, medicine, surfaces with controlled wettability, etc. [1,4,6,7,8]. Among the organics, highly conjugated heteroaromatic macrocyclic compounds such as porphyrins and their synthetic analogues (phthalocyanines) have shown remarkable properties, such as intense absorption in the visible domain, structural flexibility, and compatibility with plastic substrates [9,10,11]. These organic materials are of interest in different application areas: optoelectronics (non-linear optical materials [12], photovoltaic cells [13], and organic light-emitting diodes [14]) and medicine (photodynamic therapy [15,16]). Moreover, metal phthalocyanines are cheap, nontoxic, and stable (chemically and thermally) semiconductors that can be used in various devices [3,7,11,17]. The inorganic nanostructures to be embedded into an organic matrix in order to obtain nanocomposite thin films must feature an adequate morphology and size [18]. From inorganics, zinc oxide (ZnO) and copper oxide (CuO) can be easily obtained (in large quantities) as nanostructures with a controlled morphology and size by various wet and dry approaches that involve raw readily available materials and inexpensive equipment [18,19,20,21,22]. The fabricated metal oxide nanostructures are used in electronics, food packaging applications, or water treatment [18,20,23,24].

Organic–inorganic systems based on phthalocyanine (or porphyrin derivatives) and inorganic semiconductors were investigated for their suitability in photovoltaic cells [25,26,27], chemical gas sensors [7,28,29], photocatalysis [3], the biomedical field [30], etc. Although different wet preparation methods (sol–gel [31], Langmuir–Blodgett [32], cathodic electrodeposition [25], etc.) were used for preparing such hybrid films with suitable properties for a target application, spin-coating remains the most accessible technique for the deposition of nanocomposite films [7,33,34]. However, spin-coating requires a highly concentrated solution (of which even 95% is lost during the deposition process) and substrates characterized by a certain wettability and size make this method inaccessible in some conditions [7,35,36]. Moreover, the properties (such as the boiling point, vapor pressure, and evaporation rate) of the solvent implied in the fabrication of the mixed layers have a great impact on their morphology and roughness [37]. Thus, films deposited by spin-coating from blend solutions containing solvents with a high vapor pressure display an increased surface roughness because the evaporation occurs faster [38], whereas films deposited from slowly evaporating solvents feature a smooth surface [39]. 

Matrix-assisted pulsed laser evaporation (MAPLE) is a laser-processing technique initially developed to deposit soft materials (especially biomaterials), which is currently extended to the fabrication of composite layers [18,40,41]. Thus, various types of substrates with different wettability were covered by MAPLE from solutions with a low concentration of material (frequently 1–5% mass concentration) [41,42,43]. Several deposition parameters can be tuned in order to obtain films with suitable properties, the most important being: (i) the solvent involved in the preparation of the solution that is further frozen to fabricate the MAPLE target and (ii) the laser fluence used during the deposition process [44,45]. The selection of the solvent must be made in correlation with its properties and the laser wavelength, meaning that the solvent must completely dissolve the organic compound and absorb the laser energy [44]. Hence, many solvents, such as dimethyl sulfoxide (DMSO), chloroform, toluene, o-xylene, pseudocumene, chlorobenzene, 1,2-dichlorobenzene, 1,2,4-trichlorobenzene, or tetrahydrofuran, were used in the MAPLE deposition for obtaining films characterized by a suitable morphology, which were further integrated into optoelectronic devices [44,46,47,48,49,50,51]. Thus, poly [2-methoxy-5-(2′-ethylhexyloxy)-1,4-phenylene vinylene] (MEH-PPV), a luminescent polymer used frequently in organic light-emitting diodes (OLEDs), was deposited from toluene or tetrahydrofuran by MAPLE [49]. Poly 3-hexylthiophene (P3HT) is another polymer suitable for organic photovoltaics that was deposited by MAPLE from o-xylene [50]. In addition, polymeric films based on regio-regular poly [3-(4-octyloxyphenyl) thiophene] (POOPT) were fabricated by MAPLE from chloroform [51]. These studies emphasized that the obtained MAPLE films preserved the chemical structure and the optical properties of the organic raw materials, their morphology being influenced by the substrate temperature. 

In the MAPLE process, similar to the behavior noted in the spin-coating related to the vapor pressure parameter, smooth films are deposited when low-vapor-pressure solvents are used [44]. It has to be mentioned that, even if the surface of the films prepared from a low-pressure solvent (e.g., DMSO) is smoother than that of the films prepared from high-pressure solvents (e.g., chloroform), the MAPLE films can contain solvent traces due to the formation of solvent droplets at the laser–target interaction [52]. 

In this context, the present study was focused on the MAPLE deposition and characterization of nanocomposite layers based on macrocyclic compounds (zinc phthalocyanine (ZnPc) and 5,10,15,20-tetra(4-pyridyl)21H,23H-porphyrin (TPyP)) with different types of conduction (ZnPc-p and TPyP-n) and metal oxide nanoparticles (ZnO or CuO) also with different types of conduction (ZnO-n and CuO-p). ZnPc is a well-known metal phthalocyanine characterized by high absorption properties, and is more soluble in different solvents than other similar phthalocyanine compounds (e.g., copper phthalocyanine) [53]. TPyP is a porphyrin that presents absorption in the visible part of the solar spectrum and can be easily obtained in thin-film form [54]. 1,4-dioxane is involved as a solvent (for the first time) in the preparation of MAPLE targets, this compound being a good solvent for ZnPc and showing good absorption at the laser wavelength (248 nm) used during the MAPLE process [53,55]. Although, 1,4-dioxane was recently utilized as a solvent in the deposition of polycaprolactone through a material jetting technique for biomedical applications [56] and as a co-solvent (together with methanol) in the deposition of perovskite films by spin-coating for photovoltaic cell applications [57], to our knowledge, there is no report on using 1,4-dioxane as a solvent in the MAPLE deposition.

## 2. Experimental Section

The organic compounds ZnPc, TPyP, and 1,4-dioxane were purchased from Sigma Aldrich and used as received. The chemical reagents Zn(CH_3_COO)_2_, Cu(CH_3_COO)_2_, NaOH, and ethanol were purchased from Merck and used without further purification. Metal oxide nanoparticles were synthesized by modifying the precipitation procedures described in the references [58,59]. Thus, under vigorous stirring, an aqueous solution of 0.25 M NaOH was added in an aqueous solution of 0.1 M Zn(CH_3_COO)_2_ for ZnO while an ethanol solution of 0.036 M NaOH was added in an ethanol solution of 0.018 M Cu(CH_3_COO)_2_ for CuO. After 1 h at 70 °C, the white (ZnO) and black (CuO) precipitates were collected by centrifugation, washed with distilled water, and dried at room temperature. The morphological, structural, and optical properties of the prepared metal oxide nanoparticles were investigated using a Zeiss Merlin Compact field emission scanning electron microscope, a Bruker D8 Advance set-up (in a Bragg-Bretano geometry) with Cu Kα1 (λ = 1.4506 Å) monochromatized radiation, and a Perkin Elmer Lambda 45 UV-Vis spectrophotometer equipped with an integrating sphere. 

In the next step, a laser with excimer (KrF*, Coherent, CompexPro 205, λ = 248 nm, τFWHM ~25 ns) and 1,4-dioxane were involved in the MAPLE deposition of the organic and composite films. The reference layer based only on the organic compounds (ZnPc:TPyP, in 1:1.5 ratio) was prepared from a solution with 3% weight/volume concentration in 1,4-dioxane. In the case of composite layers, the inorganic nanoparticles (ZnO or CuO) were dispersed in the organic solution mixture (ZnPc:TPyP), keeping the concentration (3% weight/volume) in 1,4-dioxane constant. Practically, 15% or 25% of the amount of TPyP was replaced by an amount of ZnO nanoparticles in order to prepare the P1 and P2 samples, while the same percentage (15% or 25%) of ZnPc was replaced by an amount of CuO nanoparticles for obtaining P3 and P4 samples. Thus, depending on the component weight ratio in the MAPLE deposited layers (ZnPc:TPyP:ZnO:CuO), the investigated samples were labelled as follows: P0 (1:1.5:0:0), P1 (1:1.275:0.225:0), P2 (1:1.125:0.375:0), P3 (0.85:1.5:0:0.15), and P4 (0.75:1.5:0:0.25). The same experimental parameters were employed in all MAPLE deposition: 300 mJ/cm^2^ laser fluence, 70 000 number laser pulses, 20 Hz repetition rate, and 5 cm target–substrate distance. In the same deposition cycle, the organic and composite layers were deposited on glass and silicon substrates for structural, morphological, and optical measurements and on indium tin oxide (ITO) covered with a thin layer of poly(3,4-ethylenedioxythiophene)-poly(styrenesulfonate) (PEDOT:PSS, 40 nm) substrates for evaluating the potential application of the developed structures in the optoelectronic domain. More details about the MAPLE process and the deposition of PEDOT:PSS film on ITO/glass substrates by spin-coating are provided in the reference [46]. 

Further, the organic and nanocomposite layers deposited by MAPLE were assessed from morphological, vibrational, optical, and electrical point of view. The thickness was estimated as an average media of three scans in different points by an Ambios Technology XP 100 profilometer. The morphology and the elemental composition were evaluated using a Zeiss Gemini SEM 500 field emission scanning electron microscope equipped with an energy-dispersive X-ray analysis Quantax Bruker XFlash detector 610 M accessory and a Nanonics 4000 Multiview atomic force microscope. The infrared spectra were collected in the 700–1700 cm^−1^ domain with an IRTracer-100 spectrometer, the UV–Vis spectra in the 250–850 nm range by a Thermo Scientific Evolution 220 Spectrophotometer, and the photoluminescence (PL) spectra in the 450–750 nm domain (λexc = 435 nm) using an FL 920 Edinburgh Instruments spectrometer with a 450 W Xe lamp excitation and double monochromators on both excitation and emission. 

In order to perform the electrical measurements, on top of the organic or composite layers deposited on ITO/PEDOT:PSS, lithium fluoride (LiF, 1.5 nm) and aluminium (Al, ~100 nm) films were deposited through shadow masks by vacuum thermal evaporation using a Tecuum AG, VCM600-V3-80 set-up. The role of the LiF film is to improve the electron injection. The substrates were kept at room temperature and the pressure in the deposition chamber was 1.6 × 10^−6^ mbar. Hence, the electrical and photo-electrical behavior of the fabricated structures were investigated from current density–voltage (J-V) measurements carried out in dark and under illumination (AM 1.5, incident power density equal to 100 mW/cm^2^), all tests being performed in air. The experimental set-up was formed by a Keithley SourceMeter 2400 model, a Newport Oriel monochromator, and a Newport Oriel solar simulator controlled by a computer, the working interface being a homemade one based on LabVIEW 7.1 software (National Instruments, Austin, TX, USA).

## 3. Results and Discussion 

**Figure 1:**
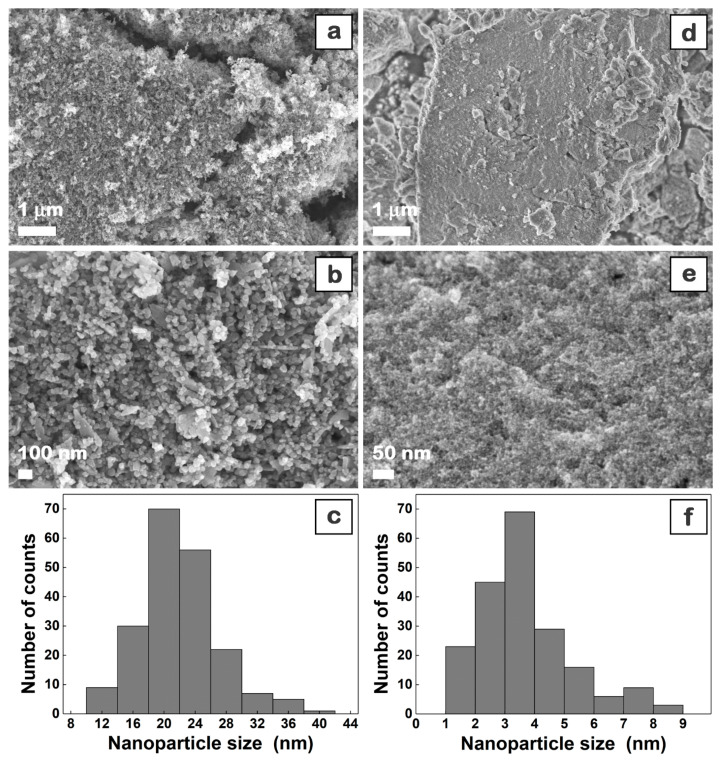
FESEM images (at two magnifications) and particle size distribution histograms of the chemically synthesized ZnO (**a**–**c**) and CuO (**d**–**f**) nanoparticles.

The morphological, structural, and optical properties of the chemically synthesized inorganic powders were firstly evaluated. The FESEM images (Figure 1) disclose that ZnO (Figure 1a,b) and CuO (Figure 1d,e) powders are formed by quasi-monodispersed particles with sizes of ~20 nm and ~5 nm, respectively. Further, the particle size distribution histograms of the metal oxide nanoparticles were obtained using the ImageJ 1.53t software, these disclosing a relatively homogeneous size distribution.

**Figure 2:**
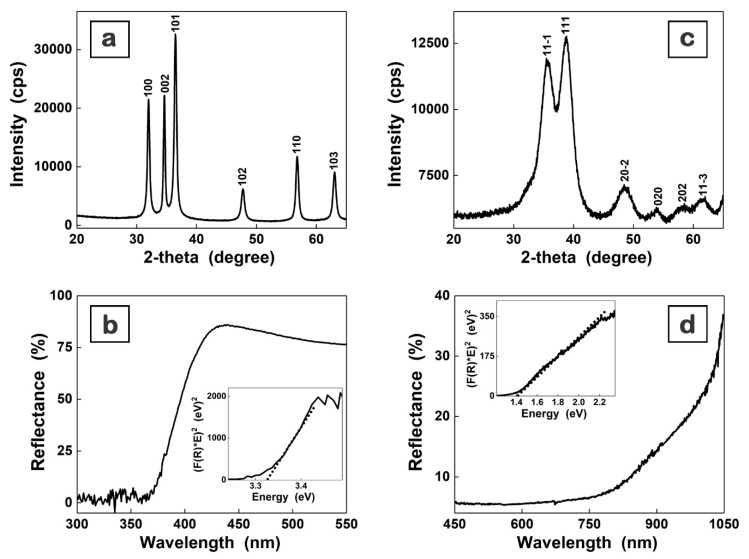
XRD patterns (**a**,**c**) and reflectance spectra (**b**,**d**) of the chemically synthesized ZnO (**a**,**b**) and CuO (**c**,**d**) nanoparticles.

The XRD patterns (Figure 2a,c) reveal the main peaks corresponding to the Miller indexes of the reflecting planes assigned to the hexagonal wurtzite ZnO structure (Figure 2a), ICDD 00-035-1451, and monoclinic CuO structure (Figure 2c), ICDD 00-048-1548. The mean crystallite size (D) of the metal oxide samples was estimated at ~17 nm for ZnO and ~2 nm for CuO using the Debye–Scherrer equation D = Kλ/βcosθ, where K = 0.9 (shape factor), λ = 0.154 nm (wavelength of the incident CuKα radiation), θ = the Bragg angle, and β = FWHM (full width at half maximum of the most intense diffraction peaks). In the optical reflectance spectra (Figure 2b,d), a strong decrease can be observed below ~400 nm and ~900 nm due to the band-to-band transition in ZnO and CuO, respectively, the band gap value being estimated at around 3.32 eV for ZnO and 1.42 for CuO by plotting [F(R)*E]^2^ versus photon energy (E), where F(R) is the Kubelka–Munk function, with F(R) = (1 − R)^2^/2R, and R is the observed diffuse reflectance (insets Figure 2b,d). Both band gap values are in agreement with those previously reported for these two semiconductors [21,60,61].

These results confirm that the prepared metal oxide nanoparticles have adequate properties for their embedding in organic–inorganic nanocomposite films with a thickness of ~100 nm.

**Figure 3:**
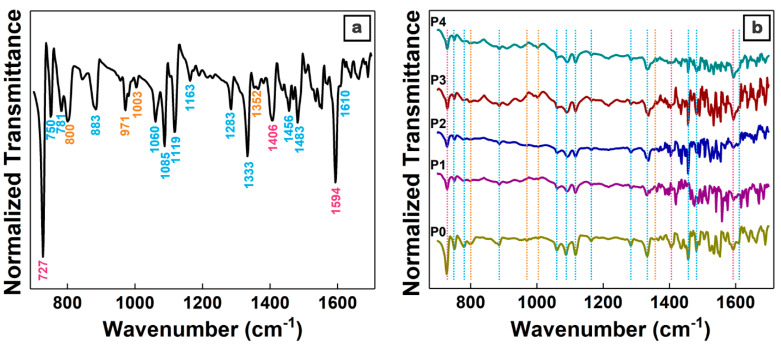
FTIR spectra of the reference film prepared by drop-casting from P0 solution (**a**) and P0–P4 films deposited by MAPLE (**b**) on silicon. Color legend of peaks and lines: magenta—both organic components, ZnPc and TPyP; blue—ZnPc; orange—TPyP.

Subsequently, the organic and composite films deposited by MAPLE were assessed by infrared spectroscopy in order to analyze the preservation or damage of the chemical structure of the organic components during the MAPLE process. FTIR measurements were carried out both on MAPLE deposited films and on films obtained by drop-casting from the solution containing only the organic compounds (the same solution used in the preparation of the target involved in the deposition of the P0 sample by MAPLE). In this way, an accurate evaluation can be achieved, taking into account that the specific vibration bands of each organic component can be more easily identified in the FTIR spectra of the thicker film deposited by drop-casting (Figure 3a) than in the FTIR spectra of the MAPLE deposited films (Figure 3b). The phthalocyanines are synthetic analogues of porphyrin [9]; hence, absorption bands observed at approximately 727 cm^−1^ (C-H bond out-of-plane deformation), 1406 cm^−1^ (isoindole stretch), and 1594 cm^−1^ (stretching of the C=C bond in benzene from ZnPc or in pyridyl from TPyP [54,62]) can be assigned to both organic compounds. Specific phthalocyanine vibration bands are observed at approximately 750 cm^−1^ (C-H bond in-plane deformation), 781 cm^−1^ and 883 cm^−1^ (benzene breathing), 1060 cm^−1^, 1085 cm^−1^, 1119 cm^−1^, 1163 cm^−1^, 1285 cm^−1^ (C-H bond bending), 1333 cm^−1^ (in-plane stretch of pyrrole), 1456 cm^−1^ and 1483 cm^−1^ (isoindole stretch), and 1610 cm^−1^ (C=C bond stretch in benzene) [62]). Specific TPyP vibration bands are noted at approximately 800 cm^−1^ (C-H bond vibration in pyrrole), 971 cm^−1^ and 1003 cm^−1^ (vibration of the C-N bond and relaxation of the porphyritic ring, both specific to the porphyrin base), and 1352 cm^−1^ (stretching of the C=N bond), respectively [63].

Although, in the FTIR spectra of the films deposited by MAPLE, the absorption bands of ZnPc and TPyP are less intense due to the film thickness ranging from 60 nm to 105 nm, their presence confirms that the chemical structures of both organic components are preserved during the MAPLE deposition.

**Figure 4:**
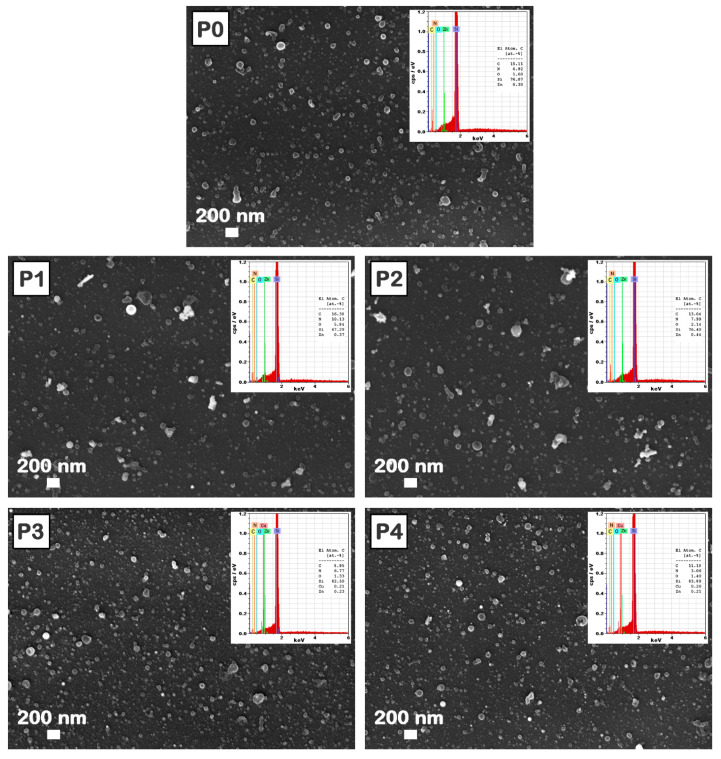
FESEM images of the P0–P4 films deposited by MAPLE on silicon. Insets: EDX spectra and atomic percentages of the elements in the corresponding samples.

**Figure 5:**
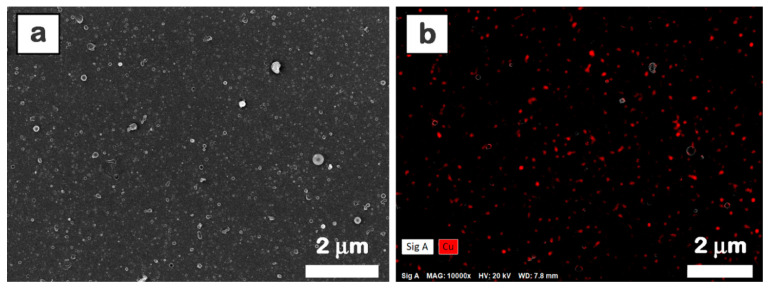
FESEM image (**a**) and EDX mapping (**b**) only for Cu element of P4 film deposited by MAPLE on silicon.

**Figure 6:**
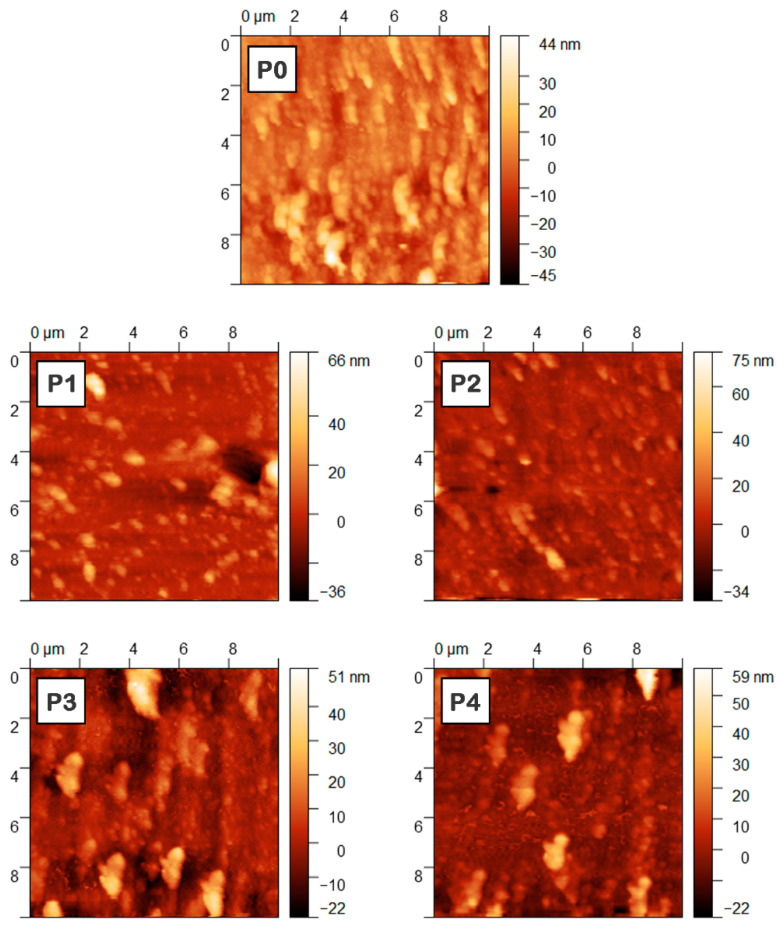
AFM images of the P0–P4 films deposited by MAPLE on ITO/PEDOT:PSS (10 μm × 10 μm).

Usually, the morphology and the thickness of the layers involved in the fabrication of devices have a major impact on their performance. FESEM images (Figure 4), EDX maps (Figure 5), and AFM images (Figure 6) of the MAPLE deposited films reveal that these are uniform and continuous with some aggregates on their surface, with a morphology specific to the MAPLE films [63]. 

The FESEM images (Figure 4) disclose that the aggregates are randomly distributed on the layer surface. As expected, the presence of the metal oxide nanoparticles increases the size and number of the aggregates, which tend to form clusters, a similar effect also being observed in the case of films deposited by spin-coating [64]. Moreover, the FESEM images (Figure 4) revealed that the P1 and P2 samples present larger clusters than P3 and P4 samples due to the presence of ZnO nanoparticles, which have larger sizes than those of CuO nanoparticles (Figure 1). Thus, the globular morphology characteristic to the MAPLE deposition [42,46] together with the particularities linked to the metal oxide nanoparticle size and components ratio result in films with surfaces containing aggregates that have a clusterization tendency. The presence of the metal oxide nanoparticles in the composite films deposited by MAPLE is proved by the EDX spectra (Figure 4 insets), the signals corresponding to C, N, O, Zn, and Cu (elements contained by both organic and inorganic components) being identified. For all EDX spectra, the most intense peak is assigned to the Si signal, silicon substrates being used in the deposition process. In the composite films, the increase in the amount of ZnO or CuO leads to an increase in the Zn or Cu atomic percentage in P1–P2 and P3–P4, respectively. The difference noted in the Zn signal can be explained by taking into consideration the following aspects: the ZnPc amount is the same in the P0–P2 samples and is lower in the P3–P4 samples (due to the addition of CuO instead of ZnO), so the Zn atomic percentage increases from the P0 film (based only on ZnPc:TPyP) to the P1 film and further to the P2 film due to the addition of ZnO, and decreases from the P3 film to the P4 film due to the increase in the added CuO amount in these samples.

Further, the distribution of the metal oxide nanoparticles in the composite films was assessed. Taking into account that the presence of Zn peak can be related to both ZnPc and ZnO, and is not relevant for the presence of only ZnO nanoparticles, the FESEM image (Figure 5a) and the corresponding EDX map (Figure 5b) are given for the P4 film, this sample containing the highest amount of CuO nanoparticles. 

Hence, the EDX map illustrates a uniform distribution of the Cu element on the entire surface of the composite layer, confirming that the metal oxide powder was well dispersed in the organic mixture during the preparation of the target and was further uniformly transferred in the composite layer.

The influence of the addition of inorganic nanoparticles in the organic mixture on the surface and thickness of the MAPLE deposited layers was further explored. Thus, the roughness parameters (root mean square (RMS), roughness average (Ra)) were evaluated based on the AFM images (Figure 6), their values and the layer thickness being summarized in Table 1. 

It can be noted that the thickness of the composite layers decreases in comparison to that of the reference organic film. As already mentioned, in the case of composite layers, the inorganic nanoparticles were dispersed in the organic solution mixture, keeping the concentration in the solvent constant so that the organic content is reduced in these samples due to the addition of the inorganic nanoparticles. Usually, the insertion of metal oxide nanoparticles within the organic active layer leads to an increase in the thickness of the hybrid layer [65]. In the present case, a possible explanation for the thickness decrease takes into account the presence of TPyP in the organic mixture (ZnPc:TPyP). Compared to the planar molecule of ZnPc, TPyP is a mesosubstituted porphyrin in which the pyridyl groups can be rotated out of the porphyrin plane, a conformational adaptation effect on the deposition substrate of this molecule being reported [66]. In addition, the porphyrin compounds can be adsorbed on the surface of metal oxide nanoparticles, modifying their orientation on the substrate surface and affecting the molecular packing [67]. 

Concerning the roughness parameter, the RMS value obtained for the P0 sample is very small in comparison to those of the organic films based on the same small molecule compounds deposited by MAPLE in very similar conditions using DMSO [63]. 1,4-dioxane, the solvent used in this study, features a high vapor pressure (3.7 kPa at 20 °C [57]). After the arrival and deposition on the substrate, the solvent rapidly evaporates, leading to the supersaturation of the precursor mixture and formation of a large number of nuclei, resulting in smoother and homogeneous films than those prepared from conventional solvents such as dimethylformamide or DMSO. 

A low roughness is still noted for the composite layers, even if the RMS parameters are slightly increased. Interestingly, although the P2 sample contains the highest ZnO amount, a variation of 1.2 nm is obtained between its roughness and that of the P0 sample based only on organic components. In the case of this sample, the thickness of the layer and the roughness parameter value suggest that the presence of the ZnO nanoparticles does not have a major impact on these parameters, these being well distributed within the composite film. In addition, the roughness values of the samples containing CuO nanoparticles are similar to those recorded for the layers based on ZnO nanoparticles. Although the roughness values of the composite layers are slightly increased in comparison to that of the reference organic layer, they are still smaller than the values reported for the hybrid layers based on organic compounds and metal oxide nanoparticles fabricated by spin-coating [64,68]. In addition, the roughness of the composite films prepared by MAPLE is smaller than the roughness recorded for the hybrid films based on poly [2,6-(4,4-bis-(2-ethylhexyl)-4H-cyclopenta [2,1-b;3,4-b0]dithiophene)-alt-4,7-(2,1,3-benzothiadiazole)] (PCPDTBT) and CdSe nanoparticles deposited by another laser technique, resonant infrared MAPLE (RIR-MAPLE) [47], which is usually regarded as an alternative to MAPLE for fabricating smoother films.

**Figure 7:**
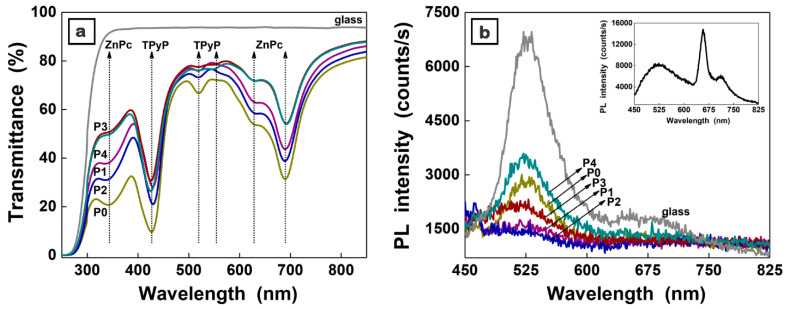
UV–Vis spectra (**a**) and PL spectra (**b**) of the P0–P4 films deposited by MAPLE on glass. Inset: PL spectrum of TPyP film obtained by MAPLE on glass.

The optical properties of the MAPLE deposited films were evaluated from the UV–Vis spectra (Figure 7a) and PL spectra (Figure 7b). Both organic materials are characterized by absorption bands in the visible part of the solar spectrum, the shape of the UV–Vis spectra being almost the same for all investigated samples. Hence, the absorption bands assigned to ZnPc are identified as follows: a Soret (B) band at a lower wavelength between 310 nm and 390 nm, with the maximum at ~340 nm, and a Q band with its specific splitting in two maxima at 630 nm (π-π^∗^ transition [69]) and 690 nm (excitonic transition [70]).

Macrocyclic compounds with a highly conjugated π-electron system as the phthalocyanines, the porphyrine compounds such as TPyP present also the B and several Q bands [54,71]. Thus, in the P0 sample, the TPyP signature consists of: an intense narrow B band with a maximum at ~430 nm (allowing electronic transitions between π-π* orbitals of the porphyrin ring [72]) and a Q band with a maximum at ~520 nm (transitions to the first excited singlet state [72]). Generally, TPyP presents several Q sub-bands [73], the symmetry of the molecule being responsible for their number [72]. Thus, another barely visible maximum at ~550 nm can be identified in P3 and P4 samples that contain the same TPyP amount as the P0 sample. Due to the strong ZnPc absorption, the other two maxima at ~590 nm and ~640 nm characteristic to TPyP cannot be clearly identified in the UV–Vis spectra.

With regard to the emission properties, all PL spectra were dominated by the emission band peaking at ~530 nm due to the glass substrate. However, in the case of the TPyP film deposited by MAPLE on glass, the PL spectrum (Figure 7b inset) is present beside the substrate emission, its specific split emission band with two maxima at ~660 nm and ~710 nm in agreement with other data reported for the TPyP layers deposited by vacuum evaporation or MAPLE (using DMSO as solvent) [54,63]. This strong emission is not identified in the PL spectra of the organic and composite films, most probably due to a quenching effect that takes place between the components of the layers. In addition, the emission associated to ZnPc at ~700 nm, which is usually a weak emission [63], cannot be observed in the PL spectra of the investigated samples. It has to be noted that, due to their small amount, the absorption and emission bands of the metal oxide nanoparticles are not visible in the UV–Vis and PL spectra of the composite films.

Regarding the recombination of the charge carriers, this takes place most probably by non-radiative processes, taking into account that the photoluminescence measurements suggest that the emission is quenched in the composite films.

**Figure 8:**
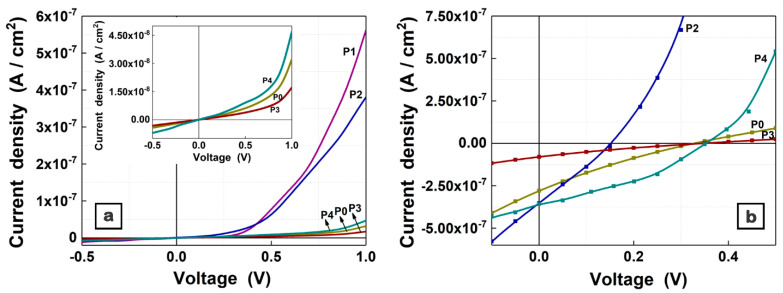
J-V characteristics in dark (**a**) and under illumination (**b**) in A.M. 1.5 conditions of the structures based on organic (P0) and composite (P1–P4) films deposited by MAPLE.

**Figure 9:**
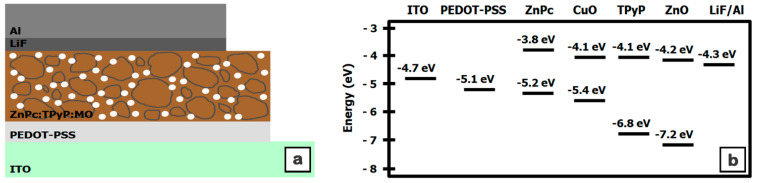
Schematic representation of the structure based on macrocyclic compounds:metal oxide (MO) particles nanocomposite thin films (**a**) and the energy levels diagram of the structure components (**b**).

The J-V characteristics (Figure 8) were acquired in the dark (Figure 8a) and under illumination (Figure 8b) on the structures based on the MAPLE deposited nanocomposite films. In addition Figure 9 shows a schematic representation of the fabricated structures based on ITO/PEDOT:PSS/nanocomposite/LiF/Al and the energy level diagram of the constituent materials [46,54,74,75]. The J-V characteristics recorded in the dark (Figure 8a) are strongly asymmetrically non-linear, suggesting that the structures have rectification properties. In the following, the electrical parameters recorded for the structures based on composite layers (P1–P4) are compared to those obtained on the reference layer based only on the organic compounds (P0), taking into account that the electrical parameters are influenced by the addition of metal oxide nanoparticles in the active layer. Thus, an increase in the dark current density value (~3 × 10^−8^ A/cm^2^) was recorded for the structure prepared only with organic compounds (ZnPc:TPyP) in comparison to the value already reported for the structures based on the same mixture but with the active layer deposited from the other solvent (DMSO) [63]. A typical diode behavior (Figure 8a) is observed for the structures containing ZnO nanoparticles. In our case, a current density of at least one order higher, for an applied voltage of 1 V, was noted for the structures developed with ZnO nanoparticles, compared to the reference cell. The result can be explained taking into consideration the higher electron mobility of the metal oxide nanoparticles (~2 × 10^−3^ cm^2^V^−1^s^−1^ [76]) with respect to that of the replaced n-type organic compound (10^−4^ cm^2^V^−1^s^−1^ for the porphyrin derivatives [77]). Even the dark current density value obtained for the structure based on the P3 composite film is lower compared to that recorded for the structure containing the P0 film, and a small improvement was achieved for the structure fabricated with a higher amount of CuO nanoparticles, P4. A study focused on the hybrid films containing poly 3-hexylthiophene (P3HT), [6,6]-phenyl-C61-butyric methyl ester (PCBM), and CuO nanoparticles (at various ratios) reported that the highest obtained mobility was of approximately ~5 × 10^−4^ cm^2^V^−1^s^−1^ for a certain amount of inorganic nanoparticles [68]. A possible explanation for the current density value acquired in the case of the P3 sample can be linked to the presence of some defects that can affect the carriers’ mobility.

Further, the J-V characteristics of the structures developed with MAPLE prepared layers were acquired under illumination (Figure 8b), taking into account that these films feature significant absorption in the visible and that the electrical characteristics recorded in the dark suggest that they can find application in the photovoltaic cell field. In addition, relevant electrical parameters, such as the short-circuit current density (J_SC_), open circuit voltage (V_OC_), and maximum power (P_max_), were calculated.

Excepting the structure based on the P1 film, all of the structures exhibited a photovoltaic effect. The J_SC_ value interpolated from the J-V curve of the structure prepared with the organic film (P0 sample) was 2.8 × 10^−7^ A/cm^2^, while the V_OC_ value was 0.32 V and the P_max_ was 2 × 10^−8^ W. A small increase in the J_SC_ value (3.5 × 10^−7^ A/cm^2^) and a lowering in the V_OC_ value (0.15 V) and P_max_ (1.3 × 10^−8^ W) were recorded for the P2 sample. This result is in accordance with other studies reported on structures containing ZnO nanoparticles, the addition of a large amount of inorganic nanostructures leading to a decrease in the V_OC_ value [46,74,78]. This effect was explained taking into consideration the agglomeration of the nanoparticles that can favor the recombination of electron–hole pairs within the bulk active layer.

Usually, the V_OC_ value for the organic cells is provided by the difference between the donor highest occupied molecular orbital (HOMO) and the acceptor lowest unoccupied molecular orbital (LUMO) (Figure 9b). In the present study, for the ZnPc (donor) and TPyP (acceptor), we considered the HOMO to be at 5.2 eV [46] and the LUMO to be at 4.1 eV [54], respectively. In addition, the review from reference [79] reported that the V_OC_ value can be directly or indirectly affected by various parameters, such as the charge carriers recombination, light intensity, morphology, electrode work function, donor/acceptor interface area, crystallinity, charge carriers density, etc. Thus, for the structures based on organic films deposited by MAPLE, one of the factors that can influences the V_OC_ value is the crystallinity of the layers, a parameter that can be improved by applying some thermal treatment to the near-amorphous organic layers deposited by MAPLE [46].

In the case of samples containing CuO nanoparticles, a lowering in the J_SC_ value (7.9 × 10^−8^ A/cm^2^) and in the P_max_ (4.1 × 10^−9^ W) was obtained for the P3 sample, whereas an increase in the J_SC_ value (3.6 × 10^−7^ A/cm^2^) and in P_max_ (4.5 × 10^−8^ W) was achieved for the P4 sample. These results can be explained by considering the following aspects: the insufficient amount of CuO nanoparticles that can contribute to the photocurrent; the reduced number of the photogenerated excitons, which, in turn, is due to the smaller quantity of the principal donor (ZnPc in this case); and/or the defects generated during the deposition of the film. With regard to the increase in the J_SC_ value in the P4 sample, taking into consideration that both P3 and P4 films present similar absorption properties and roughness, the higher amount of CuO nanoparticles in the P4 sample can result in larger interfaces that favor the exciton dissociation process. A recent report emphasized that the presence of CuO nanoparticles does not significantly change the V_OC_ in comparison to the organic film based on P3HT:PCBM [80]. A similar effect was observed in this study: regardless of the CuO nanoparticles amount added to the organic mixture, the V_OC_ values recorded for the P3 and P4 samples, 0.35 V and 0.36 V, respectively, are very close to that obtained for the P0 sample containing only the ZnPc:TPyP mixture.

Concerning the influence of the type and amount of the metal oxide nanoparticles on the electrical properties of the investigated structures, it has to be mentioned that the quantity of inorganic nanoparticles was added in such a manner as to preserve the same donor:acceptor ratio (1:1.5), where the amount of CuO in the P4 sample is practically smaller than that of ZnO in the P2 sample. In the case of the samples based on CuO nanoparticles, the maximum content at which the electrical properties can be altered has probably not been reached.

Ergo, the structures based on nanocomposite films deposited by MAPLE are sensitive to the amount of metal oxide nanoparticles added in the organic mixture, an improvement in the electrical parameters being achieved only for the photovoltaic structures containing the suitable organic and inorganic components in an optimum ratio.

## 4. Conclusions

Organic and nanocomposite films based on macrocyclic compounds (ZnPc:TPyP) and metal oxide (ZnO or CuO) nanoparticles were prepared by MAPLE using 1,4-dioxane as a solvent. The FTIR spectra confirm the preservation of the chemical structure of the organic compounds in the MAPLE deposited films. The UV–Vis spectra disclose the characteristic absorption bands of ZnPc and TPyP while the PL spectra reveal a quenching effect of the specific intense emission band of TPyP. The FESEM images evidence agglomerated particles on the composite film surface but their presence has no significant impact on the roughness of the deposited MAPLE layers. The AFM images proved that the films deposited by MAPLE using 1,4-dioxane as a solvent are characterized by a low roughness (an essential feature of films with applications in optoelectronic devices) compared to similar films deposited from other solvents. The addition of metal oxide nanoparticles influences the electrical properties of fabricated composite structures depending on their conduction type and amount. An increase in the current density value (recorded in the dark) was obtained for composite films that contain a higher amount of nanoparticles. The J-V characteristics recorded under illumination show that most structures developed with MAPLE deposited films present a photovoltaic effect. The best electrical parameters were obtained for the structure based on film with a higher amount of CuO nanoparticles. Consequently, by tuning the composition and ratio between the organic and inorganic components of the composite films, an improvement in the electrical properties of the structures based on such MAPLE deposited layers can be achieved, making them suitable for potential applications in the field of optoelectronic devices.

## Figures and Tables

**Table 1:** The component ratio, thickness, and roughness parameters of the MAPLE deposited films.

Sample	Component Ratio ZnPc:TPyP:ZnO:CuO	Thickness (nm)	RMS (nm)	R_a_ (nm)
P0	1:1.5:0:0	105	5.3	3.9
P1	1:1.275:0.225:0	60	8.3	5.3
P2	1:1.125:0.375:0	95	6.5	4.4
P3	0.85:1.5:0:0.15	95	8.8	6.2
P4	0.75:1.5:0:0.25	90	8.6	5.6

## Data Availability

The data presented in this study are available on request from the corresponding author.
